# Obesity Proteomics: An Update on the Strategies and Tools Employed in the Study of Human Obesity

**DOI:** 10.3390/ht7030027

**Published:** 2018-09-12

**Authors:** Afshan Masood, Hicham Benabdelkamel, Assim A. Alfadda

**Affiliations:** 1 Obesity Research Center, College of Medicine, King Saud University, P.O. Box 2925 (98), Riyadh 11461, Saudi Arabia; afsmasood@ksu.edu.sa (A.M.); hbenabdelkamel@ksu.edu.sa (H.B.); 2 Department of Medicine, College of Medicine, King Saud University, P.O. Box 2925 (38), Riyadh 11461, Saudi Arabia

**Keywords:** obesity, proteomics, LC-MS/MS, human, adipose tissue, review

## Abstract

Proteomics has become one of the most important disciplines for characterizing cellular protein composition, building functional linkages between protein molecules, and providing insight into the mechanisms of biological processes in a high-throughput manner. Mass spectrometry-based proteomic advances have made it possible to study human diseases, including obesity, through the identification and biochemical characterization of alterations in proteins that are associated with it and its comorbidities. A sizeable number of proteomic studies have used the combination of large-scale separation techniques, such as high-resolution two-dimensional gel electrophoresis or liquid chromatography in combination with mass spectrometry, for high-throughput protein identification. These studies have applied proteomics to comprehensive biochemical profiling and comparison studies while using different tissues and biological fluids from patients to demonstrate the physiological or pathological adaptations within their proteomes. Further investigations into these proteome-wide alterations will enable us to not only understand the disease pathophysiology, but also to determine signature proteins that can serve as biomarkers for obesity and related diseases. This review examines the different proteomic techniques used to study human obesity and discusses its successful applications along with its technical limitations.

## 1. Introduction

Obesity as a disease has challenged the research, academic, and healthcare communities through not only its varied pathogenesis, but also its treatment, prevention, and management modalities. The last decade has seen an exponential rise in the number of overweight and obese individuals. Obesity itself has transitioned from being a term indicating increased fat mass, mainly in the adipose tissue, to that of a disease entity that is characterized by a state of chronic low-grade inflammation. The main feature of the disease is a dysfunction of the adipose tissue, along with a disturbed profile of circulatory adipokines and elevated levels of other pro-inflammatory factors in the blood. Adipose tissue is a highly heterogeneous tissue not just in its structure and morphology, but also in its biochemical, physiological, and endocrine functions. It influences a host of metabolic and biochemical pathways, regulates total energy homeostasis, and integrates signals to and from the central nervous system and other peripheral organs through its diverse metabolite and adipokine secretome [1,2].

The use of human adipose tissue to study obesity, although significant, has been especially challenging due to its high lipid content, as has been the case with other body fluids because of their complexity. Although a wealth of knowledge has accumulated regarding obesity and adipocytes, it is well known that single approach is insufficient to understand the complex biochemical and physiological changes taking place in humans. Genomic studies have also lagged behind in this regard, as they cannot completely describe the different changes within the adipocyte proteome nor delineate its protein content [3]. This was mainly attributed to the presence of numerous posttranslational modifications, single-nucleotide polymorphisms, and alternative splicing forms of the proteins.

Studying human obesity through the use of human samples from adipose tissue, blood, or other biological fluids is pertinent to understand the molecular mechanisms and metabolic pathways leading to obesity or its associated comorbidities. This is possible with the development of high-resolution and high-throughput mass spectrometry (MS) proteomic and efficient separation techniques, which when combined with other high-throughput techniques would assure comprehensive approach to this complex disease. In this review, we highlight the different proteomic techniques that have been employed to study human obesity and its associated comorbidities.

## 2. Background of Mass Spectrometry-Based Proteomic Techniques

Mass spectrometry proteomic techniques provide us with an unbiased and technology-driven approach that has the capacity to comprehensively catalog the entire protein components within a cellular organelle, cell, or a tissue. Mass spectrometry is an ideal analytical tool for the high-throughput discovery of protein alterations in both health and disease [4]. It helps in the large-scale study of proteins within a sample, determination of protein composition, structure and function, and interactions between proteins in the cell. Proteomics has now been accepted as a key technology in biochemistry, cell and systems biology, and drug discovery. The goal of high-throughput proteomics is thus to decrease sample analysis time while increasing the depth of proteome coverage.

### 2.1. Applications of Proteomics

Generally, proteomic techniques are performed based on their research applications as either expression, functional, or structural proteomics. These approaches can be used for proteome profiling, comparative expression analysis of two or more groups of samples, localization and identification of posttranslational modifications, and the study of protein–protein interactions.

Expression proteomics is a data-centric, hypothesis-generating approach that is applied to discovery-based and targeted proteomics workflows. It generates large qualitative data sets on protein expression levels providing a global analysis of protein composition, posttranslational modifications, and the dynamic nature of expression [5,6]. Quantitative data can also be obtained through this approach by the spot pattern analysis of the relative changes in protein expression levels between two samples in response to disease or environmental stimuli. Functional proteomics elucidates biological functions of unknown proteins, defining cellular mechanisms at the molecular level and providing a detailed description of the cellular signaling pathways by revealing protein-protein interactions in the cell [7]. Structural proteomics, on the other hand, is used in targeted workflows to understand the three-dimensional shape and structural complexities of functional proteins and their associated protein complexes.

The choice of the proteomic method depends on the nature of the research and the desired end result, rather than a random selection of the technique and instrument. The proteomic workflow thus incorporates mass spectrometric analysis of the samples coupled to one or more high-resolution separation techniques to improve sample separation and overcome its complexity.

### 2.2. Proteome Analysis by Mass Spectrometry

Mass spectrometry is an analytical technique that allows for the detection of proteins by the identification of specific ionic spectra generated on the basis of their unique fragmentation patterns or mass spectrums obtained based on their mass-to-charge ratios. Identification of these experimental mass spectra and their subsequent matching to well annotated genomic databases helps to elucidate the different peptides within the sample. A few peptides, in turn, are sufficient to unambiguously characterize the proteins that are present in the sample or their differential abundance in the disease state. These platforms form the mainstay of experimental proteomics for protein analyses with a greater sensitivity, efficient throughput time, and capacity for an in-depth proteome analysis. Mass spectrometric analyses incorporate the use of an ionization technique coupled to a mass detector of choice.

Ionization is commonly achieved by soft ionization methods, such as matrix-assisted laser desorption/ionization (MALDI) [8] and electrospray ionization (ESI) [9], to measure the masses of proteins and peptides without significant fragmentation. Both of the methods can be used to analyze proteins up to >100 kDa [10].

In MALDI, the analytes are ionized with a crystalline matrix via laser pulses, whereas in ESI, they are directly ionized from a solution typically eluted from liquid chromatography (LC) columns as electrically charged gaseous ions. MALDI is usually adopted to analyze simple samples, whereas LC-ESI is used to analyze complex mixtures.

### 2.3. Types of Mass Analyzers

The most common types of mass analyzers used in clinical proteomics are time-of-flight (TOF), quadrupoles, ion trap, ion cyclotron resonance, and hybrid-sector/trap and quad/TOF mass analyzers. Selecting an analyzer is based on their mass resolving power, mass accuracy, mass range, and the linear dynamic range.

- In TOF mass spectrometers, the flight times of ions are measured over a fixed distance to match a specific mass-to-charge ratios (*m*/*z*), and the intensity of a measurement is correlated with the amount of the ion. The TOF analyzer is the fastest MS analyzer with a high resolving power that is well suited for pulsed ionization methods (e.g., MALDI), has a high ion transmission and highest practical mass range of all MS analyzers, and is able to acquire the entire spectrum from a single laser pulse event.
- The quadrupole mass analyzer is a mass filter that can be set to pass only ions of a selected mass-to-charge ratio and separates them based on their oscillating electric field (the quadrupole field). Quadruple mass analyzers are an excellent choice when developing mass spectrometry methods for targeted analysis. They have a good reproducibility and are relatively small, low-cost systems with limited resolution and they are not well suited for pulsed ionization methods and thus are used mostly with gas chromatography mass spectrometry (GC/MS) and liquid chromatography mass spectrometry (LC/MS) systems [11].
- The ion trap mass spectrometer uses ESI as the preferential ionization method. It works by trapping the ionized analytes by electric and/or magnetic fields before subjecting it to collision by selectively ejecting analytes from the ion-trapping field for detection. When compared to other mass analyzers, it has poor quantitation capabilities, has a very poor dynamic range, and is subject to space charge effects and ion-molecule reactions, but it is still sensitive enough to measure non-abundant analytes. Despite their relatively low mass accuracy, ion traps (ITs) have been commonly used to obtain the majority of proteomic data [10,12].
- Fourier transform ion cyclotron resonance-mass spectrometry (FTICR) is used for determining the *m*/*z* of ions based on the cyclotron frequency of the ions in a fixed magnetic field. It has the highest mass resolving power and best mass measurement accuracy among the current mass analyzers. Fourier transform ion cyclotron resonance-mass spectrometry has recently been applied to identify high-mass analytes, low abundance compounds and proteins in complex mixtures and to resolve species of closely related *m*/*z* ratios [13]. Coupled with high-pressure liquid chromatography (HPLC) and ESI, FTICR-MS is able to characterize single compounds (up to 500 Da) and detect the masses of peptides in a complex protein sample in a high-throughput manner. The Orbitrap analyzer (Thermo Fisher Scientific, Waltham, MA, USA), which is a variant of the FTICR-MS is currently widely used in proteomics research and it is chosen for complex proteome analysis [14].

## 3. Mass Spectrometric Platforms Used in Human Obesity

Proteomic technologies studying obesity and adipose tissue encompass the whole array of the previously mentioned methods in different combinations with each other. The most common platforms and combinations that are used to derive important biological information are MALDI-TOF-MS, ESI-LC-MS, surface-enhanced laser desorption/ionization mass spectrometry (SELDI-TOF-MS), and protein microarray [15].

- Matrix-assisted laser desorption/ionization time of flight mass spectrometry has emerged as a valuable technique for identification of compounds in the *m*/*z* range of 500 to over 100,000. The methods are relatively simple to use, have a high mass accuracy and are reasonably less affected by contaminants and solvents [12]. The optimal combination of MALDI is with a TOF mass analyzer to analyze proteins and peptides with a wide range of molecular weights [10]. A prerequisite for MALDI-TOF analysis is an initial separation of proteins that is carried out by either gel-based or non-gel-based methods. Due to its simplicity, excellent mass accuracy, high resolution, and great sensitivity, MALDI-TOF-MS has been widely adopted to identify proteins that are associated with diseases, including obesity [10].
- Electrospray ionizationis a more versatile solution-based ionization method that introduces mixtures of biomolecules into the MS instrument. The unique feature of ESI is that it allows for the rapid transfer of analytes from the liquid phase to the gas phase at atmospheric pressure in the presence of a high electric field to produce submicrometer-sized droplets, which eject charged desolvated analyte ions into the mass spectrometer for subsequent mass measurement. ESI can be used with most types of mass detection systems, including TOFs, ion traps, or hybrid TOF MS and quadrupoles. When compared with MALDI, ESI has a significant advantage in the ease of coupling to separation techniques, such as LC, which includes HPLC and ultra-high-pressure liquid chromatography (UPLC), allowing for high throughput and online analysis of peptides or protein mixtures. Electrospray ionization-quadrupole ion trap, TOF-MS has very good resolution power with the advantage of high mass accuracy for the mass determination of proteins less than 2000 [10].

## 4. Separation Techniques

Separation techniques are important as they reduce the complexity of any given proteome. They can be described as ‘offline techniques’, such as two-dimensional gel electrophoresis or ‘online techniques’, such as LC, in combination with the mass spectrometry methods. Each technique selected has its own advantages as well as disadvantages. The main strategies currently applied to proteome research utilize high-resolution complimentary separation techniques based on either electrophoresis or chromatography. These strategies involve the use of gel-based electrophoresis protein separation by 1- or 2-dimensional sodium dodecyl sulfate polyacrylamide gel electrophoresis (1-DE or 2-DE SDS-PAGE), gel-free separation techniques, and targeted techniques while using chip-based protein microarrays coupled to MS.

### 4.1. Application of Gel-Based Techniques to Human Obesity

These techniques, although labor intensive, are very important methods in separating proteins as they resolve complex protein mixtures into their different components, helping in visualization, identification, and characterization. Gel-based separation techniques, generally termed as the top down techniques, include conventional 1-DE or 2-DE SDS-PAGE, and the more robust 2-DE difference gel electrophoresis (DIGE) technique.

#### 4.1.1. One-Dimensional Gel SDS-Polyacrylamide Electrophoresis 

This technique is relatively simple to perform and is reproducible; thus, it is most commonly used in the characterization of proteins as it has the limited resolving power of a 1-DE gel. It relies on the separation of proteins based only on their molecular weights. This technique is good for the analysis of proteins within simple samples, containing hydrophobic proteins or very large membrane proteins up to 2000 kDa. We could identify only one study by Leggate et al. who used 1-DE SDS PAGE as their method of separation prior to coupling it directly to MALDI TOF. In their study, they determined the inflammatory and prominent proteomic changes in both plasma and adipose tissue between overweight and obese males after high-intensity intermittent training [16].

One-dimensional gel separation has been used more often as the first step of sample fractionation followed by LC while using nano LC (nLC), UPLC, or HPLC linked to ESI and different mass detectors, such as Orbitrap (Thermo Fisher Scientific) or hybrid linear ion trap (LTQ)-FTICR. Subsequently, many investigators have used this technique in studying obesity and its associated proteomic changes in different tissues and with different physiological or comorbid conditions. The skeletal muscle proteome was studied while using 1-D SDS-PAGE gel separation followed by nLC-ESI-tandem mass spectrometry(MS/MS) Orbitrap to study the role of obesity and roux-en-Y gastric bypass surgery on the human skeletal muscle proteome [17]. One dimension gel electrophoresis was also used by coupling it to HPLC-ESI-MS/MS to study the changes between skeletal muscle from obese and morbidly obese women [18] that showed an increase in proteins essential for the maintenance of cellular energy charge and the glycolytic enzymes glyceraldehyde-3-phosphate dehydrogenase and aldolase A in obese/overweight and morbidly obese women. The same technique was used by Hwang et al. to determine the abnormalities in patients with obesity and type 2 diabetes [19] who identified an increase in mitochondrial and three cytoskeletal proteins of myocytes, α actinin 2, myozenin-1, and desmin in insulin resistance. Lindinger et al. studied abdominal omental adipose tissue biopsies separated by 1-DE gel coupled to anion-exchange chromatography, followed by comparison and identification by MALDI-TOF/TOF [20] and identified reduced levels of mitochondrial proteins in the adipocytes from the obese. The subcutaneous adipose tissue, on the other hand, was studied by Lehr et al. while using 1-DE SDS-PAGE/LC ESI-MS, ion trapping, and two-dimensional SDS-PAGE/MALDI-TOF/TOF MS to profile the human adipokinome identifying 44 proteins as adipokines and validating the increase in heme oxygenase-1 levels in obese subjects [21]. The subcellular organelle proteome, including that of the endoplasmic reticulum in adipose tissue of obese, insulin-resistant individuals, was studied by Xie et al. by coupling 1-DE gel to HPLC-ESI-MS/MS and using LTQ-FTICR MS to demonstrate an increase in endoplasmic reticulum stress-related proteins and genes [22]. Lazar et al. used nLC-MS/MS coupled to an LTQ-Orbitrap to study the proteome of adipocyte exosomes to discern the mechanism linking obesity and cancer and found an increase in proteins that are related to fatty acid oxidation in the obese that amplified tumor migration [23]. Kolmeder et al. studied the colonic metaproteomic signatures of active bacteria and the host between obese, non-obese and morbidly obese fecal samples from 29 subjects, separated by 1-DE gel electrophoresis and characterized them using reverse phase (RP) LC-MS/MS [24,25]. SELDI-TOF MS was used by Alvarez-Llamas et al. for the profiling, identification, and characterization of proteins while using LC-MS/MS after SDS-PAGE fractionation using ESI- quadrupole-quadrupole-TOF-MS/MS from human visceral adipose tissue (VAT) secretome [25].

#### 4.1.2. Two-Dimensional Gel Electrophoresis 

Two-dimensional gel electrophoresis has been the mainstay of proteomics and it is the most widely used separation tool in expression proteomics when comparative analysis is desired. It consists of two steps: isoelectric focusing (IEF) that separates proteins based on their isoelectric points, and SDS-PAGE, which separates the proteins according to their molecular weights. The map of protein spots obtained can be considered to be the protein fingerprint or proteomic signature of the sample. Visualized protein spots (by use of different dyes, such as Coomassie blue, silver nitrate, and fluorescent dyes) are then excised and digested for further identification. 2-DE SDS-PAGE is mostly coupled to MALDI TOF mass spectrometry, and as such, many studies on human obesity have used this technique independently or followed by ESI–LC-MS/MS using quadrupole time of flight analyzers (QTOF), Orbitrap, or FTICR.

Two-dimensional electrophoresis coupled to MALDI TOF was used to identify proteins that are differentially expressed in visceral white adipose tissue between obese/overweight and control groups and to study the role of the identified proteins in adipocyte differentiation [26]. Obesity is a well-known causative factor for cardiovascular disease; 2-DE coupled to MALDI TOF was used by Moreno et al. to study the anatomical heterogeneity in the proteome of human subcutaneous adipose tissue to identify a close relationship between obesity and cardiovascular disease [27]. Total body adiposity is known to correlate well with the amount of adipose tissue around the myocardium and the coronary arteries, the so-called epicardial adipose tissue. This idea was used to analyze the epicardial and subcutaneous adipose tissue in the obese patients to reveal differences in proteins that are involved in oxidative stress and in lipid transport of epicardial adipose tissue associated with coronary artery disease [28,29]. Ahmed et al. carried out a proteomic analysis of human adipose tissue after rosiglitazone treatment, showing coordinated changes to promote glucose uptake using ESI–LC–MS/MS coupled to a high-capacity ion trap mass spectrometer [30]. Similarly, the same method was used to study the human mammary adipose tissue proteome to identify extracellular and intracellular signaling components of mammary subcutaneous adipose tissue (SCAT) adipose tissue and its interstitial fluid in high-risk breast cancer patients [31]. Subcutaneous adipose tissue from abdominal biopsies was also used to study the physiologic effects of caloric restriction on the adipocyte-enriched proteome of overweight/obese subjects [32]. Boden et al. identified and measured an increase in endoplasmic reticulum stress-related proteins and genes in adipose tissue of obese and insulin-resistant individuals [33,34]. The method was also used to study the fasting whole adipose tissue protein profile between low and high-fat oxidizers [35], as well as adipocytes differentiated from obese human mesenchymal stem cells [36] and the stromal-vascular fraction of adipose tissue obtained from subcutaneous and visceral fat depots [37]. The liver proteome was also explored for the identification of proteins altered by type 2 diabetes mellitus in obese subjects [38] and in liver biopsies between obese and non-obese subjects using 2-DE MALDI-TOF-MS and nLC-ESI-MS/MS in an attempt to obtain novel biomarkers and promising targets for drug development in obesity [39]. These studies showed deregulation of proteins involved in mitochondrial function and methionine metabolism in liver of obese diabetic patients and a deregulation of enzymes that are involved in fatty acid β-oxidation, ketogenesis, pyruvate metabolism, gluconeogenesis, and oxidative stress, among others, in the liver biopsies from human obese subjects.

In addition to its application in studies using tissues, 2-DE MALDI-TOF-MS was also applied successfully for proteomic analysis of plasma in obese and overweight prepubertal children [40].

Adipogenesis or adipogenic differentiation is a contributing factor to the obesity epidemic and was also studied through a proteomic approach. DeLany et al. used primary cultures of human adipose-derived adult stem cells (ADAS) from subcutaneous liposuction aspirates as an in vitro model of adipogenesis using 2-DE and capillary liquid chromatography interfaced to an ESI-MS/MS Q-TOF [41]. Lee et al. on the other hand, used obese human mesenchymal stem cells to look at protein expression that is inherent to adipogenic differentiation by 2-DE MALDI-TOF [36]. Renes et al. also used 2-DE linked to a CE ESI-MS/MS Q-TOF micromass spectrometer to study human ADAS cells isolated from subcutaneous liposuction aspirates of six non-diabetic, healthy donors [42]. 2-DE coupled to affinity chromatography was used to purify human serum-derived protein complexes associated with fat-derived hormone adiponectin to identify their nature by nLC-ESI-MS/MS [43].

#### 4.1.3. Two-Dimensional-Difference Gel Electrophoresis 

Two-dimensional-difference gel electrophoresis is a variation of 2-DE that relies on pre-electrophoresis labeling of samples distinctly with one of the fluorescent CyDyes (Cy2, Cy3, and Cy5). This provides an advantage over the conventional 2-DE as it allows for multiplexing of samples (experiment vs. control, disease vs. normal) into the same gel, enables the detection of subtle changes in protein expression levels, and produces accurate and reliable results with a minimal loss of proteins as no post-electrophoretic processing is needed. 2D-DIGE is routinely coupled to MALDI-TOF mass spectrometry. The peptide masses resulting from digestion are determined by mass spectrometry while using peptide mass fingerprinting or tandem mass spectrometry for de novo sequencing. A majority of the studies dealing with clinical obesity have utilized the 2D-DIGE separation techniques that are linked to MALDI-TOF analyzers. Proteome profiling analyses of white adipose tissue from healthy human and/or obese patients have been done to elucidate the molecular mechanisms of obesity or obesity-related diseases.

The differences between SCAT and VAT proteomes in human obesity were studied while using 2D-DIGE MALDI-TOF by an untargeted proteomic approach by Insenser et al. [44]. The VAT proteome between obese and non-obese patients was again studied by Perez-Perez et al. who described the presence of attenuated metabolism as a hallmark of obesity by comparative proteomic analysis of human omental adipose tissue. They also used the same technique to identify differences between two different adipose tissue depots, namely, omental and SCAT, in their biochemical and metabolic properties; they subsequently uncovered suitable reference proteins for expression studies in human adipose tissue relevant to obesity [45,46,47]. Murri et al. presented data obtained from a proteomic analysis of VAT in pre-obese patients with and without type 2 diabetes while using 2D-DIGE MALDI-TOF/TOF, and suggested an increase in proteins involved in cytoskeletal function and structure, oxidative stress, inflammation, and retinoid metabolism in the pre-obese diabetic group [48]. While Corton et al. studied the paired omental and subcutaneous human adipose tissue proteome and described methods to improve resolution while using 2D-DIGE MALDI-TOF/TOF at an alkaline and wide-ranging pH. They also studied the omental adipose tissue biopsies that were obtained from morbidly obese women with or without polycystic ovary syndrome (PCOS) to examine the possible involvement of visceral adiposity in the development of PCOS. Their study revealed differential expression pattern of proteins that are involved in lipid and glucose metabolism that included Apoprotein A1, annexin V, and glutathione S-transferase in PCOS patients [49,50]. Obesity is known to affect pregnancy and in many circumstances leads to gestational diabetes. Oliva et al. conducted proteomic expression profiling while using 2D-DIGE MALDI-TOF/TOF through the use of omental adipose and placental tissue obtained from lean and obese pregnant women during cesarean section to identify the proteins that are associated with gestational diabetes and determine the effects of preexisting maternal obesity on the placental proteome [51]. Proteomic profiling of SCAT from obese individuals was also carried out by our group to study the effects of obesity and aging [52] and to understand the effects of protein differences on the metabolic pathways of lean, overweight and obese individuals [53]. Apart from this, the 2D-DIGE MALDI-TOF approach was used to profile VAT, the more metabolically active fat depot, extensively. The proteome expression of VAT and/or SCAT was studied between metabolically healthy and unhealthy obese phenotypes to gain knowledge of the molecular factors and pathways responsible for progression to the latter to understand the role of proteasome dysfunction associated with oxidative stress and insulin sensitivity in human obesity [54] and to understand the effects of bariatric surgery-induced weight loss on adipose tissue [55]. Similarly, the VAT and SCAT proteomes that were obtained from morbidly obese male and female patients during bariatric surgery were explored to understand the effects of androgens on AT [56], as well as on the muscle proteome of severely obese women with androgen excess when compared to severely obese men and non-hyperandrogenic women [57].

The two-dimensional-difference gel electrophoresis coupled to MALDI-TOF technique was also used to examine other biological fluid, such as the urinary proteome of obese patients to understand the characteristics of different weight loss strategies while using calorie restriction and bariatric surgery [58]. The endometrium of obese and overweight women with recurrent miscarriages was used to find evidence of any endometrial defects with obesity [59]; human amniotic mesenchymal stem cells underwent proteomic analysis to understand the effects of maternal obesity on the risk of obesity and/or obesity-related diseases in the offspring [60]. In addition, the proteome of the human spermatozoa was also analyzed to detect any associated proteomic changes with obesity or diabetes and revealed 15 differentially expressed proteins that included prolactin-induced protein, outer dense fibre protein 1, spermatogenic glyceraldehyde 3-phosphate dehydrogenase, and semenogelin 1 [61]. Although MALDI-TOF is the preferred downstream mass spectrometry choice after 2D-DIGE, we found one instance where 2D-DIGE was coupled to nano HPLC/ESI-MS/MS to analyze the proteome of both human SCAT and SVF cells versus mature adipocytes from obese patients [62].

#### 4.1.4. Disadvantages of Gel-Based Proteomics

Although gel-based proteomics is a widely used technique, it has disadvantages. Not only is this method labor intensive and time-consuming, but it also fails to efficiently separate proteins of either, very high molecular weight, very low molecular weight, proteins with an alkaline pH, or very hydrophobic proteins. An example of this is membrane proteins (they are hydrophobic, and their extraction requires strong detergents that are not compatible with first dimension separation as they precipitate) that are known to precipitate during IEF and are almost never observed. Thus 2-DE fails to visualize all of the proteins in a complex sample and can visualize only 30–50% of the entire proteome, thereby prompting the use of gel-free techniques.

### 4.2. Applications of Gel-Free Approaches to Human Obesity

These approaches utilize non-gel-based methods for the fractionation of samples. They have a higher yield of protein substrates and peptide products by avoiding intercalation in the 2D gel matrix. Various methods for peptide purification have been devised, including off-gel electrophoresis, capillary electrophoresis (CE), liquid chromatography—labeled or unlabeled, and a combination of techniques, such as multidimensional protein identification, cation-exchange chromatography, and RP-LC.

#### 4.2.1. Off-Gel Electrophoresis Mass Spectroscopy

This is a gel-free approach that fractionates proteins and peptides according to their pH. The separation is done by IEF using a pH gradient, and the proteins or peptides are recovered from the liquid phase and directly analyzed by LC-MS/MS without further clean-up or processing. This is done using a gel-free multi-compartment electrolyzer electrophoretic device or by adapting the off-gel IEF to a multi-well format, which leads to efficient protein fractionation and identification. The off-gel method was used by Kim et al., who applied the technique to study the fractions of pooled VAT samples coupled to LC-MS/MS in drug-naive early type 2 diabetes mellitus (T2DM) patients [63]. Wang et al. also used off-gel electrophoresis in plasma samples to screen for obesity-related protein biomarkers and found lower levels of adiponectin and increased levels of C-reactive protein and phosphatases in the obese group when compared to the controls. The same method was also used for the screening of plasma samples from obese and non-obese patients with T2DM, followed by further separation and identification by a nano HPLC-Chip-MS/MS system [64,65].

#### 4.2.2. Capillary Electrophoresis Mass Spectroscopy 

This is another gel-free proteomic analytical technique that is highly sensitive, robust, and reproducible. Electrophoretic separation of peptides in CE is done according to the charge and size of the proteins by applying high voltage and analyzing the proteins in the mass spectrometer. Obstructive sleep apnea (OSA) is a common complication of obesity, having a substantial negative impact on a patient’s quality of life and increasing the risk of cardiovascular disease. A discovery profiling using CE coupled to a microTOF MS was used by Seetho et al. to characterize the urinary proteomes between severely obese patients with and without OSA and also between severely obese subjects with OSA receiving continuous positive airway pressure treatment when compared to those without OSA [66,67].

#### 4.2.3. Liquid Chromatography Tandem Mass Spectrometry Approach

The LC-MS/MS approach is a contemporary method of electrophoresis, which is commonly referred to as the bottom-up proteomic or the shotgun proteomic technique. The complex nature and large dynamic range of proteomes require the use of partial purification, depletion of high-abundance proteins, and selective enrichment prior to LC. The technique couples weak or strong cation exchange (SCX) LC with RP LC, so that peptides are separated first on the basis of their charge or their hydrophobicity. The disadvantage of this method is that it requires an immense amount of time and computing power to deconvolute the data obtained. In addition, considerable time and effort may be expended in the analysis of uninteresting proteins. The LC-MS/MS approaches include label-free methods and labeled methods for the identification of the proteome.

A. Liquid Chromatography-MS/MS—label-free methods

Label-free quantitative proteomics is a gel-free method applied to quantitatively analyze protein samples, especially the low-abundance proteins in mixed samples by using spectral counts or measuring peptide ion intensities as a read-out of protein abundance [68]. This method has found greater applicability and it has been widely used in clinical studies. Relative quantification is achieved through observing the measured ratios of quantified proteins and the number of tandem mass spectra of a peptide. The most widely used shotgun approach has been reverse phased liquid chromatography RP-LC mass spectrometry (RP-LC-MS/MS) with (HPLC or UPLC while using ESI or nano ESI (nESI) as the ionization source.

Plasma samples were analyzed by the label-free shotgun approach while using UPLC-Q-TOF MS for metabolic profiling of plasma between overweight/obese and lean men [69]. The human plasma proteome was also explored by nano-electrospray ion source with a UPLC coupled to an Orbitrap in the large dietary intervention (DiOGenesDIOGENES) to determine the effects of sustained weight loss on the human SCAT proteome [70]. Similarly, serum samples from African-American women with metabolically normal and abnormal obesity were analyzed by µRPLC-MS/MS coupled to a linear ion trap mass spectrometer to identify proinflammatory and lipid biomarkers mediating metabolically healthy obesity [71].

Abu-Farha et al. also applied shotgun proteomic profiling approaches while using RP-nLC-MS/MS coupled to an Orbitrap on peripheral blood mononuclear cells that were isolated from lean and obese men to identify and quantify proteins that are modulated by exercise [72]. Plasma proteins were interrogated by Dayarathna et al. after two-step prefractionation while using immunodepletion and multi-lectin affinity chromatography. The prefractionation steps allowed for the removal of abundant proteins by two complementary methods, followed by LC-MS/MS coupled to a linear ion trap mass spectrometer for the analysis of obesity and associated diabetes and hypertension [73]. The RP-LC-MS/MS method was also used to profile post-translationally modified proteins, such as carbonylated plasma proteins as potential biomarkers of obesity-induced type 2 diabetes mellitus between lean and obese patients with or without T2DM by [74]. An nESI-HPLC linear trap quadrupole XL (LTQ XL) ion trap mass spectrometer was also used to analyze SCAT and VAT proteomes in diabetic and non-diabetic patients with morbid obesity [75]. Circulating human adipocyte-derived extracellular vesicles from the SCAT of obese subjects undergoing elective bariatric roux-en-Y surgery were analyzed by nanoscale RPHPLC-MS/MS while using nanospray ionization coupled to a triple TOF hybrid mass spectrometer to identify the novel markers of metabolic stress [76].

In addition to whole adipose tissue, the label-free quantitative proteomics approach using nLC/MS-MS coupled to a QTOF MS was used to carry out a comparative analysis of the secretory proteome of obese human adipose stromal vascular fraction cells during adipogenesis [77]. The same method was coupled to an LTQ Orbitrap hybrid MS to study the secretomes of subcutaneous and omental preadipocytes from obese subjects to study the visceral white adipose tissue expansion and macrophage accumulation associated with metabolic dysfunction [78]. Proteomic secretome analysis of SCAT was performed by LC-MALDI TOF/TOF to study the regulation of monocytes by SCAT secretomes from obese/non-obese patients with heart failureusing a cell culture method [79]. A fast protein liquid chromatography–tandem mass spectrometry (MS/MS) proteomics approach was used with the fractionated secretome obtained by co-culturing preadipocytes with primary subcutaneous and visceral adipocytes or tissue explants from obese patients. Proteins that were measured by this label-free quantification were used to study the crosstalk between adipocytes and preadipocytes. The study identified a spectrum of factors that either positively or negatively affected adipocyte formation [80]. Reverse phase-HPLC ESI coupled to Orbitrap MS was also used to carry out the proteomic analysis of spermatozoa between patients with obesity-associated asthenozoospermia and clinically healthy individuals to identify the obesity-associated proteomic changes that could potentially affect sperm quality and motility [81].

B. Liquid Chromatography-MS/MS—labeled methods

These approaches use different labeling techniques that are highly suitable for LC-MS/MS to accurately quantitate proteomes, particularly in combination with stable isotope labeling approaches, such as stable isotope labeling with amino acids in cell culture (SILAC), isotope-coded affinity tags (ICAT), isotope-coded protein labels (ICPL), or isobaric tags for relative and absolute quantification (iTRAQ). The major advantage of labeling strategies is that individual protein/peptide pools are differentially tagged so that they can be multiplexed for subsequent fractionation and MS analysis [82].

Stable isotope labeling with amino acids in cell cultures typically use heavier and lighter isotopes of carbon (^13^C) or nitrogen (^15^N) to differentially label different samples that are later pooled together before mass spectrometry analysis. Peptides derived from the different samples can be distinguished due to their mass differences. Stable isotope labeling with amino acids in cell culture is a simple, inexpensive, and accurate procedure that can be used as a quantitative proteomic approach in any cell culture system. This method has been extensively used in cell culture and animal model experiments, although its use has not been widely used in human studies.

In addition to stable isotope labeling, metabolic labeling and chemical labeling are other effective ways to uniformly incorporate isotopic tags into proteins prior to mass spectrometry. Isotope-coded affinity tags is a proteomic method that is used for analysis of proteins in complex biological specimens. Isotope-coded affinity tags peptide labeling uses biotin as the affinity tag and cysteine as the reactive amino acid to differentiate between two populations of proteins while using reactive probes that differ in isotope composition, i.e., heavy reagent and light reagent. Mass Spectroscopy is then used to reveal the ratio of the isotopic molecular weight peaks that differ by 8 Da, and this gives a measure of the relative amounts of each protein from the original samples [82]. Limitations of the method include nonspecific binding to the streptavidin affinity matrix and multiple subsequent reactions at the same site. Isotope-coded affinity tags provides limited sequencing information that makes protein identification challenging. Isotope-coded protein labels is a variant of ICAT that uses an efficient amine labeling strategy that was developed to increase sequence information from protein and peptide samples by accounting for post-translational modifications. Chemical labeling through ICPL has also been made available by multiplex proteomic analysis. This multiplexed approach enables the comparison of multiple samples, three in this case, within one analytical run, with accuracy and precision that were comparable to that of the binary analyses [83]. This technique was used by Lecube et al. to carry out the proteomic analysis of cerebrospinal fluid from obese and non-obese women with idiopathic intracranial hypertension to assess the hypothalamic control on body weight regulation. Isotope-coded protein-labeled tryptic digests were analyzed on an ion trap mass spectrometer coupled to an nHPLC system [84].

Isobaric tags for relative and absolute quantitation reagents are a set of multiplexed amine-specific stable isotope reagents which consist of a reporter group based on N,N-dimethylpiperazine, a mass balance carbonyl group and a peptide-group. This method has remarkable efficiency and resolution, being capable of distinguishing between isotopes of similar mass, based on differences in their nuclear binding energy. Multiplexed iTRAQ combined with LC-MS/MS was conducted by Miao et al. to reveal the differentially expressed proteins that may predict development of childhood obesity, through a comparative proteomic analysis of umbilical vein plasma from normal and gestational diabetes mellitus patients. They identified 36 differentially expressed proteins between the two groups, including phosphatidylcholine-sterol acyltransferase phospholipid transfer protein and Rho guanine nucleotide exchange factor 11, which are known to exhibit an inhibitory effect on embryo size and lipid metabolism [85]. Al-Daghri et al. analyzed the whole serum proteomes of age-matched nondiabetic overweight and obese females while using a similar multiplex design with pooled biological and technical replicates. To bypass the basic limitations of immunodepletion-based strategies, they carried out subproteome enrichment by size-exclusion chromatography, followed by iTRAQ 2D-LC-nESI-FTICR-MS analysis [86]. Cominetti et al. on the other hand, used immuno-affinity depletion of 14 abundant plasma proteins to determine the proteomic profiles of 1000 plasma samples collected for the multi-centered Diet, Obesity and Genes project (DiOGenes study). A highly automated mass spectrometry-based proteomic workflow comprising of isobaric tandem mass tags6-plex labeling with RP-LC-MS/MS analysis coupled to a hybrid linear ion trap-Orbitrap was employed [87]. The plasma proteome profiles of the DiOGenes cohort was also used to identify proteins associated with weight loss and maintenance and explore their relationship to body mass index, fat mass, insulin resistance, and sensitivity at baseline and after combined weight loss/maintenance phases [88]. More recently, the impact of aging and T2DM on adipocyte mitochondria from obese patients was studied while using iTRAQ-labeled peptides and high-resolution nLC-MS analysis coupled to an Orbitrap Fusion Tribrid MS [89].

Comparison of isotope-labeled amino acid incorporation rates (CILAIR) is a newly developed quantitative method; it allows for the detection of low abundance secreted proteins and it provides a quantitative method to study tissue secretomes for application with differentiated cells and tissues and it is suitable for use in combination with tissue culture. It provides information on protein synthesis rates and facilitates protein origin validation. The CILAIR method allows for quantitative assessment of changes in protein secretion without the need for 100% label incorporation and it serves as a good alternative to SILAC [90]. Use of CILAIR is not limited to adipose tissue but it should also be applicable to other tissues and differentiated cell types in culture. The technique was used by Roelofsen et al. to quantitatively assess the changes in protein secretion and in hormonal regulation within the human adipose tissue secretome as a whole rather than in adipocytes alone, preserving the crosstalk between the different components of the tissue [91]. Roca-Rivada et al. recently used the CILAIR-based secretome analysis of obese visceral and subcutaneous adipose tissues to reveal distinctive location-specific secreted proteins; their differential secretion made it possible to study the obese adipose tissue microenvironment, including extracellular matrix remodeling and inflammatory status of the different fat depots [92].

### 4.3. Targeted Proteomics

Targeted proteomics deals with the identification of known proteins or a panel of proteins through MS strategies. These target proteins are selected by the discovery phase of the research, and the analysis of these proteins can be conducted by selected reaction monitoring or multiple reaction monitoring assays. Although this strategy has been applied to the study of animal models and cell lines, its use in human studies is limited.

Another technique that finds applicability in human obesity is SELDI-TOF-MS, which can quantify selected proteins between two different clinical states of the disease and has high-throughput capability. Surface-enhanced laser desorption/ionization is an adaptation of MALDI-TOF-MS that uses laser ionization along with surface-modified target plates for both the specific and non-specific binding of analytes before the mass analysis. The samples are usually incubated onto the chip surface, followed by washing and determination of the proteomic composition typically with TOF MS. Its features include a higher sensitivity of detection, accuracy of quantification, and capability to generate reproducible patterns in different laboratories. Surface-enhanced laser desorption/ionization is a powerful tool that overcomes purification and separation of proteins prior to mass spectrometry analysis. A major distinguishing feature of SELDI is its applicability for biological samples (like blood and urine) that are complex mixtures and its improved resolution of integral proteins, not just of peptide fragments. The SELDI-TOF-MS technology is capable of identifying both single protein biomarkers and their expression patterns [93].

This technique was successfully used to study the serum proteomic profiling in obese patients to characterize patients with chronic liver diseases undergoing bariatric surgery while using liver histological lesions [94] and investigate the relationship between obesity and breast cancer diagnosis and/or progression [95]. Lamy et al. performed salivary sample profiling using SELDI-TOF-MS to study the changes in the salivary proteome between morbidly obese women who had undergone bariatric surgery, morbidly obese women who did not undergo bariatric surgery, and normal weight women to identify potential mechanisms related to the development of obesity [96]. Range et al. on the other hand, studied the saliva samples using SELDI-TOF-MS analysis to identify proteome modifications in obese patients with gingival inflammation; and the higher susceptibility of obese patients to periodontal diseases [97]. Alvarez-Llamas et al. used SELDI-TOF-MS to profile the VAT secretomes and monitor changes in the dynamic range of the samples if the highly abundant contaminating proteins were removed more efficiently [25]. Major drawbacks of the system, however, include the inability to detect proteins that are tightly bound to the plate surface or are low molecular weight proteins (<20 kDa), and the inability to identify proteins after subsequent recovery.

## 5. Conclusions

We have highlighted the most common MS technologies with an emphasis on applications to clinical studies on obese patients. Mass spectrometry has so far been used to study obesity and the associated comorbid conditions while using a number of different biological samples, like adipose tissue, muscle tissue, saliva, blood, urine, and cell extracts. However, these studies have been mainly exploratory or discovery-based, leaving a large gap wherein the identified proteins can become clinically relevant. The reasons for the discrepancy between basic and clinical research are in part due to the samples heterogeneity and analytical complexity and variety. Thus, in clinical studies, obesity and its complications still need to be further explored through the use of not one but a combination of these technologies. Although the field of proteomics offers a great deal of promise, there is a need to validate these findings and determine their reproducibility for continued use in diagnostic and clinical medicine.
